# Retraction: Enhanced electrocatalytic activity and durability of highly monodisperse Pt@PPy–PANI nanocomposites as a novel catalyst for the electro-oxidation of methanol

**DOI:** 10.1039/d1ra90150h

**Published:** 2021-09-30

**Authors:** Laura Fisher

**Affiliations:** Royal Society of Chemistry Thomas Graham House, Science Park, Milton Road Cambridge UK advances-rsc@rsc.org

## Abstract

Retraction of ‘Enhanced electrocatalytic activity and durability of highly monodisperse Pt@PPy–PANI nanocomposites as a novel catalyst for the electro-oxidation of methanol’ by Özlem Karatepe *et al.*, *RSC Adv.*, 2016, **6**, 50851–50857. DOI: 10.1039/C6RA06210E.

The Royal Society of Chemistry hereby wholly retracts this *RSC Advances* article due to concerns with the reliability of the data in the published article.

The two high resolution transmission electron micrograph insets in Fig. 2 that represent Pt@PPy–PANI NPs polymer composites are identical. In addition, these insets are duplicated and scaled versions of the high-resolution transmission electron micrograph insets in Fig. 1 in an *International Journal of Hydrogen Energy* article,^1^ and in Fig. 2 in a *Journal of Cluster Science* article,^2^ by the same author group representing different nanoparticles or synthetic methods. Fig. 1 in the *International Journal of Hydrogen Energy* article^1^ represents Pt(0)/DPA@GO NPs and Fig. 2 in the *Journal of Cluster Science* article^2^ represents Pt(0) NPs. The authors claim that this was a mistake and provided replacement data for consideration. However, an expert reviewed the author’s response and concluded that it did not satisfactorily address the concerns, and that the replacement figure did not fully support the conclusions. Given the significance of the concerns about the validity of the data, the findings presented in this paper are no longer reliable.

Sinan Eriş and Fatih Sen oppose this retraction. Handan Pamuk, Yunus Yıldız, Özlem Karatepe and Zeynep Dasdelen were contacted but did not respond.

Signed: Laura Fisher, Executive Editor, *RSC Advances*

Date: 23^rd^ September 2021

## Supplementary Material
